# Examine Facilitators and Barriers to Return to Work (RTW) for Employees with Common Mental Disorder (CMD) Symptoms: A Multi-Stakeholder Qualitative Study

**DOI:** 10.3390/ijerph23060792

**Published:** 2026-06-12

**Authors:** Nandini Khatter, Sapna Chotai, Giouliana Kadra-Scalzo

**Affiliations:** Psychological Medicine, Institute of Psychiatry, Psychology and Neuroscience, King’s College London, London SE5 8AF, UK

**Keywords:** return to work, anxiety, depression, stress, employment, mental health

## Abstract

**Highlights:**

**Public health relevance—How does this work relate to a public health issue?**

- Common mental disorders (CMDs) represent one of the most pressing public health and employment challenges, affecting one million working individuals every year.
- Mental ill health is one of the main drivers of sickness absence at work.

**Public health significance—Why is this work of significance to public health?**

- Returning to work is a pivotal phase in recovery rather than a singular endpoint; therefore, understanding how workers navigate this journey from a multi-stakeholder perspective is instrumental to facilitating this process.

**Public health implications—What are the key implications or messages for practitioners, policy makers and/or researchers in public health?**

- The study draws a contrast between the procedural understanding of RTW among HR professionals and the emotional and relational experiences among service users and employment advisors.
- Successful return to work requires more than policy compliance; it depends on the alignment of organisational culture, managerial behaviour, psychological readiness and multi-layered support systems.

**Abstract:**

Returning to work (RTW) following sickness absence due to common mental disorder (CMD) symptoms, such as anxiety, depression and stress, is increasingly recognised as a critical yet complex phase of recovery. Despite this, individuals do not always experience the process as supportive or straightforward. This study explored the factors shaping RTW by examining the perspectives of service users, employment advisors (EAs) and human resource (HR) professionals. In a qualitative study, using purposive sampling, we recruited 17 participants across the three stakeholder groups. Data were collected through semi-structured interviews and analysed using thematic analysis. The findings suggest that RTW is shaped by a dynamic interplay between individual experiences, workplace relationships and organisational structures. Participants described returning to work as an ongoing and often uncertain process, influenced by shifts in confidence, expectations of support and the extent to which workplaces were able to respond flexibly to individual needs. While some accounts reflected collaborative and supportive environments, others highlighted disconnection, misalignment and unmet expectations across stakeholders. Overall, the findings point to RTW as a negotiated process, requiring alignment between employees, managers and organisational systems. The study highlights the importance of consistent, flexible and context-sensitive approaches to support sustainable RTW following CMD-related absence.

## 1. Introduction

Common mental disorder (CMD) symptoms, such as anxiety, depression and stress, represent one of the most pressing public health and employment challenges in the United Kingdom, affecting one million working individuals between 2024 and 2025 [1,2]. Recent estimates indicate that such conditions account for more than a quarter of all sickness absence, amounting to 22.1 million working days lost in the last year and placing mental ill health ahead of many physical conditions as a driver of absence [2]. The wider economic burden is even more striking, with work-related ill health estimated to cost £16.4 billion annually through lost productivity and premature labour market exit [2]. However, framing CMDs solely in economic terms risks overlooking their profound human impact. When work is disrupted by mental ill health, individuals often experience a loss of identity, diminished self-worth, reduced social belonging and erosion of daily structure, all of which are critical to psychological well-being and recovery [3,4]. Therefore, work is widely recognised as a central component of recovery from CMDs and has been associated with improved self-esteem, reduced depressive symptoms and enhanced social integration [5,6,7].

Consequently, the process of returning to work (RTW) is increasingly viewed as a pivotal phase in recovery rather than a singular endpoint [8,9]. This highlights the importance of understanding how workers with CMDs navigate this journey as the RTW process is frequently marked by significant barriers. These include certain functional impairments caused by CMDs, such as diminished self-esteem and fear of stigmatisation [10,11]. Moreover, workplace environments themselves may exacerbate distress; psychosocial factors at work are known to not only contribute to nearly 35% of depressive episodes but also impede sustainable reintegration [12,13].

The existing RTW literature has predominantly focused on identifying quantitative predictors of successful reintegration, including symptom severity, job characteristics and demographic variables [14]. While such studies offer valuable epidemiological insights, their explanatory power remains limited, often failing to capture the subjective, relational and contextual complexities of RTW. Large-scale surveys may establish statistical associations, yet they seldom contextualise how individuals emotionally interpret their return, negotiate identity shifts or reconstruct confidence and competence following prolonged absence [15]. In response, qualitative research has begun to explore the lived experience of RTW among individuals with CMDs. For example, Nielsen and Yarker [16], using interpretative phenomenological analysis, identified diverse post-return trajectories such as ‘thrivers’, ‘survivors’, and ‘exiteers’, emphasising that RTW constitutes a dynamic and evolving process rather than a discrete event. However, such studies have largely prioritised employee perspectives within organisational contexts, often overlooking broader systemic, therapeutic, and organisational support mechanisms that significantly shape RTW experiences.

Furthermore, a critical gap persists in understanding the complex relationship between individual-level influences and the RTW transition. For instance, a systematic review [17] identified high self-efficacy and a positive attitude as key enablers of sustainable RTW among individuals with CMDs. However, this stands in contrast to other findings which suggest that people recovering from CMDs often experience diminished self-efficacy and increased insecurity regarding their ability to meet job demands upon returning to work [11,18]. This tension reveals the precariousness of RTW after CMDs. If self-efficacy is a prerequisite for successful re-entry and CMDs undermine precisely this resource, then the process of return is uniquely challenging, demanding supports that extend beyond individual resilience. These contradictory findings call for an in-depth exploration of individual experiences of a successful RTW, including how workers redefine their professional identities and how this transition impacts their self-perception. Adding to this complexity is the issue of how success in RTW is conceptualised. Lagerveld and Bültmann [19] argue that traditional metrics such as job retention or absence reduction inadequately reflect whether the RTW process is successful or sustainable. Branicki and Kalfa [20] argue for a more holistic understanding, incorporating personal values, autonomy and role alignment. These dimensions, which are best accessed through qualitative methods, are underexplored in mainstream RTW research.

In recent years, attempts have been made by governments worldwide to address the detrimental effects of CMDs. In the UK, the NHS Talking Therapies service (TTS) has been developed to enhance the delivery of and access to evidence-based psychological support for depression and anxiety disorders [21]. Within this service, employment advisors (EAs) work to support the employment needs of service users in and out of work [22,23]. This integration is a significant advancement as research shows that close guidance from employment professionals plays an important role as individuals return to employment after experiencing mental ill health [24]. Despite the increasing recognition of the role of EAs in policy and practice, their own perspectives on supporting RTW remain largely absent from empirical research.

While Joosen and Lugtenberg [24] highlight the need to adopt a multi-stakeholder perspective on RTW journeys, the role of human resource (HR) professionals in RTW processes has received limited scholarly attention [25]. HR personnel occupy a strategic position within organisations, responsible for policy design, legal compliance, employee relations and the implementation of workplace accommodations [26,27]. Their unique positioning enables them to influence organisational culture, coordinate multidisciplinary support and negotiate adjustments between employees and leadership. Despite this, HR perspectives remain underrepresented in RTW research, which has primarily focused on managerial or employee experiences [28,29]. This omission is significant, given that HR professionals frequently navigate complex structural constraints, including limited training, policy ambiguity, resource shortages, and competing organisational priorities. Understanding how HR personnel perceive and manage these challenges is therefore critical for developing organisational policies and practices that effectively support employees returning after CMD-related absence.

Taken together, these gaps point to the need for a more integrated, multi-stakeholder understanding of RTW in the context of CMDs. RTW is a socially and organisationally embedded process shaped by therapeutic support, organisational structures, leadership attitudes, workplace culture and policy frameworks. By simultaneously examining the lived experiences of service users, the professional perspectives of EAs and the strategic and operational roles of HR professionals, this study aims to generate a holistic and nuanced account of the barriers and facilitators that shape the RTW journey for individuals with common mental disorder symptoms.

## 2. Methods

### 2.1. Study Design and Participants

The study adopted a qualitative study design to examine multi-stakeholder perspectives on the barriers and facilitators of RTW for people who have experienced CMD symptoms and taken time off work. We interviewed three stakeholder groups: (1) service users of TTS who had seen an EA—eligibility criteria for this group included being aged 18 years or over, having seen an EAs in TTS; experiencing mild to moderate CMD symptoms and to have been employed in the last year; (2) EAs working in TTS—eligibility criteria for this group included being aged 18 years and having been involved in supporting RTW in any capacity; (3) HR professionals who are currently employed in a full-time HR role- eligibility criteria included being aged 18 years, living and working in the UK and having direct experience supporting employees in RTW.

### 2.2. Procedures

Service users and EAs were recruited through South London and Maudsley TTS (SLAM) using purposive sampling, following a study circular across the service. HR professionals were recruited via LinkedIn posts, HR networks, community groups and professional forums to ensure a wide reach. All interested participants were screened for eligibility based on the inclusion criteria and given an information sheet and completed the consent forms prior to being scheduled for a one-to-one semi-structured interview. Interviews took place online via Microsoft Teams Meetings to maximise accessibility and reduce participant burden. Interviews were audio and video-recorded and lasted no more than 60 min. Reflexive field notes were taken after each interview to capture contextual details and reflections from the interviewers. The study received NHS (REC Ref: 24/LO/0817) and King’s College London (LRU/DP-24/25-46479) ethical approval.

### 2.3. Data Analysis

All interviews were anonymised and de-identified and imported into NVivo for coding and analysis. Reflexive inductive thematic analysis was conducted following Braun and Clarke’s six-phase framework [30,31]. This approach was selected for its flexibility and suitability for exploring subjective, contextualised experiences of RTW. In the initial stage, transcripts were thoroughly read and re-read to become familiarised with the data and develop preliminary observations. An inductive coding approach was then applied. These codes were organised into subthemes, which were subsequently refined into broader themes through ongoing discussion and comparison across participant groups. Themes were organised into two overarching domains: barriers and facilitators to RTW, in line with the study aim. The coding was conducted by two master’s students (NK and SC) with a background in organisational psychology, who had no connections to the settings that were approached as part of this study. The third coder (GKS) was a Chartered and Coaching Psychologist with extensive background in healthcare. The analytical process consisted of repeated reading for familiarisation, systematic coding of meaningful data segments, clustering codes into candidate themes, iterative refinement and consolidation of themes and development of a final thematic structure capturing shared and divergent experiences. Reflexive memos were used to inform the subthemes and theme derivation and regular peer discussions were held to derive the final themes. Following Moore and colleagues [32] recommendations, saturation was assessed iteratively during data collection and analysis using constant comparison across interviews and thematic redundancy checks. Evidence of saturation was documented until no new themes were emerging across the final interviews, at which point data collection was concluded.

## 3. Results

A total of 17 semi-structured interviews were conducted across the three stakeholder groups: HR professionals (n = 6), EAs (n = 4), and service users (n = 7). Participant ages ranged from 24 to 57 years; 12 were female and 5 were male participants. HR participants identified as female and were employed across diverse organisational contexts, including education, manufacturing, professional services, and corporate sectors, occupying roles ranging from HR Advisor to Vice President of People Experience. Service users were employed across a range of sectors, including academia, healthcare, consultancy, events and the creative industries.

Within each domain, themes were further structured across three levels of influence: individual, interpersonal and systemic. This approach enabled both shared patterns and points of divergence across the three groups to be identified. The final themes and subthemes are presented in Table 1, integrating all perspectives. Themes were organised into two overarching domains: barriers and facilitators to RTW. In total, we identified seven main themes and 11 subthemes which mapped across the three levels of influence for barriers. We identified six main themes and eight subthemes for facilitators.

### 3.1. Barriers

#### 3.1.1. Identity Disruption

Across all perspectives, reduced self-confidence and disruption to professional identity emerged as a central barrier to RTW. Service users described a loss of self-worth and competence following prolonged absence, equating absence from work with an absence of worth, *“my confidence took a real hit, not working and feeling like what you do isn’t worth anything.”* Others expressed doubts about their capability to return, such as, *“Maybe I’m just not that good, maybe nobody wants me.”* EAs closely echoed this, observing that absence often reinforced self-doubt and disengagement. As one EA noted, *“The longer someone’s off, the harder it becomes,”* describing how time away from work entrenched avoidance and eroded confidence. In contrast, HR professionals did not foreground identity disruption to the same extent. Their accounts focused more on whether employees were ready to return in functional terms, indicating a divergence in how internal experiences were conceptualised.

#### 3.1.2. Psychological Readiness

A related barrier concerned the perceived lack of psychological readiness to return to work. While closely related to self-concept and identity disruption, psychological unreadiness is distinct in its orientation. While identity disruption reflects what has already been lost during absence (e.g., a diminished sense of professional worth), psychological unreadiness is prospective, centring on anticipatory anxiety and uncertainty about one’s capacity to meet the demands of return. Service users described anticipatory anxiety, fear of failure and concerns about coping with job demands. One participant reflected, *“Scary because, for nine months, I did not practice any skills; I have to catch up with that”* while another questioned, *“Is this it now? Is this how I’m going to feel in every role?”*. EAs similarly highlighted patterns of negative thinking and avoidance, noting that individuals often feared they had “forgotten everything” or would not be able to perform as expected. Some were described as viewing work as inherently stressful or incompatible with recovery; *“They (employees) are stuck in a bit of a mindset of ‘I should be unhappy at work’, which I don’t know, they’ve learnt that through their families or that sort of a different generation.”* While HR professionals acknowledged the need for readiness, their accounts were less focused on these internal psychological processes, reflecting a divergence in emphasis between procedural readiness and lived experience.

#### 3.1.3. Managerial Capability

Managerial capability and their confidence in implementing mental health frameworks emerged as a key barrier across all groups. HR professionals described challenges in applying policies and supporting mental health-related absence, noting that *“managers don’t feel comfortable using”* policies and that procedures were *“not explained well”.* Service users experienced these gaps more directly as lack of understanding or dismissiveness. For example, one participant reported, *“My manager is not engaging with my mental health”* while others described interactions where concerns were ignored or minimised. EAs interpreted these challenges as reflecting limited mental health literacy, noting that while employers were often aware they *“shouldn’t discriminate”* they lacked deeper understanding of triggers and symptom impact. Across accounts, there was convergence on the importance of managerial competence, but divergence in how its absence was experienced and framed. While HR professionals identified capability gaps from within organisational structures, EAs observed the same deficits from the outside through the accounts of service users.

#### 3.1.4. Managerial Disengagement

Distinct from capability and confidence gaps, which concern managers’ knowledge and skill deficits, disengagement reflects a failure of relational continuity and follow-through, suggesting that barriers to effective managerial support may arise from motivational and prioritisation failures even where some degree of competence exists. Service users described prolonged periods of no contact during absence, contributing to feelings of abandonment. One participant stated, *“They don’t care about me anymore… they are just hopeful I drop off the payroll.”* HR professionals also acknowledged that RTW processes were sometimes deprioritised, with one noting that managers *“find everything else to do, but they don’t make time for return-to-work.”* They also highlighted that some managers continued to minimise psychological distress, with attitudes such as *“stress is a choice”* or tendencies to *“sweep it under the rug.*” EAs framed these experiences as relational breakdowns, highlighting the absence of consistent communication and follow-up. While all groups recognised disengagement as problematic, service users emphasised its emotional impact, whereas HR accounts were more procedural in nature.

#### 3.1.5. Psychological Safety

At the interpersonal level, stigma and reduced psychological safety were prominent barriers. Service users frequently described fear of judgement and scrutiny from colleagues, often wondering, *“Is everyone talking about me?”* and expressing concerns about being perceived as avoiding work. Experiences of exclusion and lack of empathy were also reported, contributing to reluctance to disclose difficulties or seek support. EAs reinforced this, highlighting how reduced informal interaction in hybrid working environments limited opportunities for reassurance. One EA noted, *“Small talk is really important… to break the ice”*. HR professionals acknowledged stigma as a broader cultural issue but engaged less with its interpersonal and emotional dimensions, reflecting a divergence in experiential focus.

#### 3.1.6. Policy—Environment Misalignment

Rigid and impersonal organisational policies were identified as a key systemic barrier. HR professionals described policies as insufficiently flexible, with one participant stating, “*HR policies are like… are you a square because the policies are square? … it sort of fits, but it just doesn’t work.*” Service users reflected these issues through lived experiences of denied adjustments and disciplinary action. One participant reported, “*I didn’t get any support… I actually got disciplined instead*” while another noted, “*I asked for reasonable adjustments and I was declined.*” EAs similarly identified misalignment between policy and individual need, suggesting that policies were often applied without sufficient understanding of context. There was convergence on the existence of this barrier, with divergence primarily in how it was experienced (procedural vs. personal impact). HR professionals design and administer policy, while service users are subject to it. This power asymmetry helps explain why policies intended to support recovery can, in practice, reproduce the distress they aim to address due to the divergence in the experience of the process.

Participants described job-related pressures, including workload, ambiguity, and poor fit, as barriers to RTW. Service users highlighted the stress associated with unclear expectations and excessive demands, with one stating, “Being told to do something without any preparation… is causing me worry and stress.” EAs also noted that some individuals remained in roles that were misaligned with their needs, sometimes due to financial pressures or limited alternatives. This included being “trapped” in roles that were detrimental to well-being. HR professionals acknowledged the importance of role-specific considerations, such as stress risk assessments, but their accounts focused more on formal processes than lived strain, indicating partial divergence.

#### 3.1.7. External Environment Misalignment

External and systemic pressures, including labour market constraints and access to support, were emphasised by service users and EAs. Participants described repeated job rejection as *“a nightmare*” contributing to demoralisation and reduced motivation. Limited awareness and delayed access to EA support were also highlighted. One participant stated, “*I didn’t know it existed*” while an EA noted, “*If every client had an employment advisor on day one, that would give the situation a good chance of resolving.*” These external pressures were less prominent in HR accounts, reflecting divergence in attention to broader systemic factors beyond the organisation.

### 3.2. Facilitators

#### 3.2.1. Personal Drivers

At the individual level, motivation and personal drivers facilitated RTW. Service users described financial responsibility as a key factor, with one stating, “*I’ve got a growing baby with me… I’m the breadwinner of my family.*” Others highlighted the importance of regaining identity and purpose through work: “I *think it lifts my mood and helps me cope with my mental illness, because it’s such a happy environment. Everybody is happy, cuddling and kissing their babies and things like that.*” The social pressure to return also operated through the perceived gaze of others, demonstrating that RTW motivation is co-constructed through social expectation and the felt stigma of prolonged absence; “T*he longer you don’t have a role, the more people start asking “why haven’t you had a job? What is wrong with you?—it is the perception that you can’t be out for too long. Part of it was a slight social aspect, my parents, for example, were very understanding but would occasionally ask, ‘Have you got a job yet?’*”. Confidence emerged as a central facilitator, particularly when actively developed through EA input, “*The confidence work that I did with the EA was really, really helpful because it’s like you knew that was who I was*.” EAs similarly noted that individuals were motivated by a desire to restore routine and self-worth. HR professionals placed less emphasis on these internal drivers, focusing instead on external support mechanisms.

#### 3.2.2. Managerial Support

Although managerial capability gaps and disengagement were identified as barriers in the preceding section, managerial buy-in facilitation is characterised by active, relationally engaged support. This is a qualitatively distinct condition rather than the mere inverse of those barriers. Managerial buy-in was consistently identified as a facilitator across all groups. HR professionals emphasised structured processes such as RTW meetings and regular check-ins, with one participant explaining, “*We do a return-to-work meeting but then schedule an additional meeting to make sure how they’re getting on.*” Collaborative planning was also highlighted, with HR participants stating, “*Ask them what they believe they need to support them to return-to-work.*” Service users and EAs emphasised the relational quality of these interactions, particularly when managers were supportive, responsive and consistent. Leadership and mental health literacy were also identified as key facilitators. HR professionals highlighted the importance of competent and empathetic managers, noting, “*If you’ve got somebody that is a competent manager, I think is key.*” EAs reinforced this, emphasising the need for honest and open dialogue between employees and employers: *“Understanding why work was stressful and what it’s provoked and then going in to have those honest chats with your employer to say, ‘this really stressed me out and can that be changed?’”* Service users indirectly reflected this through accounts of positive experiences where managers were understanding and supportive. The contrast between HR’s emphasis on structured process and service users’ emphasis on relational quality points to competing constructions of what support means in practice. Where HR conceives of facilitation in terms of procedural compliance and accountability, service users understand it through the quality of human connection.

#### 3.2.3. Supportive Relationships

Supportive relationships at work and beyond were described as facilitating RTW. Service users emphasised the role of family, friends and colleagues, with one participant stating, “*Those people who really loved me… never left.*” Workplace relationships characterised by patience and encouragement were also valued, with participants noting reassurance such as “*There are no stupid questions.*” EAs similarly highlighted the importance of having “*someone in your corner*” while HR participants acknowledged the role of supportive environments, though with less emphasis on informal support networks.

#### 3.2.4. Social Inclusion

Opportunities to share experiences and normalise difficulties were identified as facilitators, particularly by service users and EAs. Group therapy and peer discussions were described as reducing isolation and validating experiences. Participants noted that hearing others express similar concerns helped reframe their own experiences, making RTW feel more achievable. This desire was observed as a form of social acceptance of their experience and a relational context in which difficulty could be normalised rather than pathologized: “*if people came together as action groups… so joining like a return to work group say; something like… 4 people that in the same sort of position as you. And maybe hold a couple of introductory meetings and then say ‘Well let’s go and have coffee on Thursday and talk about things’.*” This theme was less prominent in HR accounts, indicating divergence. Some suggested the potential value of informal RTW peer groups, akin to *“baby groups,”* where individuals could meet others *“in the same sort of position”* and form supportive bonds outside clinical settings.

#### 3.2.5. Tailored RTW Support

In contrast to the policy–employee misalignment identified as a systemic barrier, tailored RTW support represents its facilitative counterpart, indicating that organisational policies can function as either obstacles or enablers depending on the degree of flexibility and individualisation with which they are applied. Tailored RTW support emerged as a central facilitator across all groups. HR professionals emphasised phased return plans and flexible arrangements, with one participant describing a “*3-month… phased return.*” They highlighted that RTW planning must be responsive to role-specific demands, particularly in safety-critical contexts. This included stress risk assessments, medication considerations, and temporary job modifications *“We’d have to do a stress risk assessment and find out exactly what elements of the role are causing that.” “Almost like the assessment they do as part of onboarding… what medication they’re taking to make sure they’re fit to drive.”* Communication frameworks, including the use of ACAS guidance, were perceived as helpful scaffolds for navigating sensitive conversations. Collaborative planning was repeatedly emphasised as essential for empowering employees and fostering shared responsibility. Service users experienced these adjustments as enabling gradual reintegration, while EAs described them as supporting both practical and psychological readiness. There was strong convergence on the importance of individualised approaches.

#### 3.2.6. Integrated Support Systems

Access to integrated support, including EAs, therapy and occupational health, facilitated RTW. Service users valued combined therapeutic and employment support, describing it as “*very good… you can carry that through*.”; “*The therapy is good… I think coaching really helps. It’s a really good space for someone to go over all these thoughts and then put them forward for what you want from your employer*.”, positioning therapeutic support as a mediating bridge between private psychological experience and the interpersonal demands of the workplace. EAs positioned their role as providing both practical and emotional support, while HR participants acknowledged the value of external services, though with more emphasis on coordination than lived impact. EAs explicitly advocated for early involvement, noting that access to support from day one could decisively shape RTW outcomes: *“I think if if every client had an employment advisor on day one, that would give the situation a good chance of resolving”*, yet this was largely absent from HR accounts, which framed support as a response to absence rather than a proactive resource. Finally, organisational culture was identified as a key contextual facilitator. HR professionals emphasised training and awareness initiatives, while service users described the benefits of working in environments where mental health was openly discussed and understood. Knowledge around medication side effects and psychological symptoms was viewed as essential for effective support; one HR professional noted, *“You need to have knowledge; have general understanding.; More awareness around medication… and how that can affect individuals.”* EAs highlighted that organisations were often “*open to learn*,” suggesting potential for improvement. Across all groups, there was convergence on the importance of a supportive and informed workplace culture.

## 4. Discussion

This study investigated the main barriers and facilitators to RTW for individuals who have taken time off work due to CMD symptoms from multiple key stakeholders, namely individuals who have taken time off (service users), EAs and HR professionals who support RTW journeys. Overall, participants experienced RTW as a fragile and negotiated process shaped by structural constraints, interpersonal dynamics and fluctuating psychological readiness. Our findings revealed that there was a considerable convergence on specific barriers and facilitators across the stakeholder groups, such as managerial, organisational and systemic role in RTW for individuals. However, our findings also suggested that there was a considerable divergence in the significance and awareness of the impact of individual factors, such as confidence and readiness in the RTW journey for individuals, with HR professionals placing less emphasis on these experiences. This divergence reflects, in part, competing institutional priorities [33] through which readiness to return is differently constructed, procedurally and functionally by HR, and psychologically and relationally by service users and EAs.

Overall, our findings largely aligned with the existing literature on CMD-related RTW, while also offering several important extensions. The structural and interpersonal barriers identified, such as fragile self-efficacy, stigma, inconsistent managerial behaviour and rigid organisational processes mirror well-established challenges in RTW research [10,11]. These barriers are consistent with Conservation of Resources (COR) theory [34] in which CMD-related absence depletes personal resources, including professional identity, confidence and social belonging, creating cascading vulnerabilities that compound the difficulties of return. Likewise, the emphasis on managerial competence, organisational culture and psychological readiness reinforces evidence that RTW is a dynamic, relational and non-linear process [8,9,35]. However, this study also provides new insights in several areas. First, while previous research highlights reluctance to disclose CMDs [36], participants here were often willing to be open; the difficultly lay not in disclosure but in whether workplaces listened or responded appropriately. Managerial avoidance, minimisation and limited mental health literacy are not only competence gaps but also constitute relational acts with stigmatising consequences, signalling to returning employees that their experience fell outside the normative expectations of working life. This aligns with Edmondson’s [37] psychological safety framework, which locates disclosure barriers less in individual reluctance and more in the perceived safety of the interpersonal climate. The absence of psychological safety rendered formal RTW support mechanisms functionally inaccessible, since employees were unlikely to engage openly with planning processes from which they anticipated misunderstanding or scrutiny. HR professionals were aware of stigma as a broader cultural issue but engaged less with its interpersonal and enacted dimensions, showing a divergence with significant implications for how anti-stigma efforts are designed and evaluated at the organisational level. Secondly, although Nielsen and Yarker [16] identified fluctuating RTW trajectories, their focus remained largely within the workplace. The present study extends this by showing personal circumstances (e.g., family responsibilities) and external environment factors such as labour market pressures interact with organisational factors to shape RTW. Thirdly, this study is among the first to examine the role of EAs in the RTW journey for individuals with CMD symptoms, an aspect rarely captured in studies focused solely on clinical or organisational perspectives.

Our findings indicate that disruption in self-confidence and professional identity are central barriers, particularly after prolonged absence, which maps directly onto identity theory. Within Burke and Stets’ [38,39] perceptual control model, individuals continuously seek verification of their role identities; when situational meanings (e.g., being “signed off” or perceived as less capable) fail to match the identity standard, the result is heightened anxiety and depression, lowered self-esteem and diminished feelings of efficacy and authenticity [40]. Tajfel and Turner’s [41] social identity theory further posits that membership to workplace in-groups can be a source of self-esteem and belonging, so prolonged absence severs the individual from a valued social category, compounding identity loss. This dual mechanism explains why participants described reclaiming their professional identity as a primary driver of return; thus, RTW functions as an identity-restoration process. The above also provides a possible explanation for the divergence of stakeholder perspectives, where HR frames readiness procedurally while service users frame it in identity terms, thus highlighting that the two groups approach this from a different positionality (e.g., organisational role occupancy versus personal and role identity verification). Furthermore, HR case management operates through the RTW process through temporal planning such as fixed return dates, formally documented accommodations and case-closure protocols, whereas for service users psychological recovery unfolds as a non-linear, contextually contingent process. This temporal desynchronisation carries substantive relational consequences as organisational support is systematically withdrawn at the point of formal case closure, which frequently precedes the consolidation of the employee’s psychological stability and coincides with the period of greatest recurrence risk [42]. While HR and managerial involvement matters, the rhythm of organisational disengagement has in itself a relational aspect, which could shape recovery outcomes independently of the intentions of any individual actor within the system.

Our findings further indicated that the disruption produced by CMD-related absence operates not merely at the functional level but at the level of vocational identity. Work is a primary vehicle for identity construction, social belonging and the organisation of self-concept [3,4,6]. Therefore, its interruption produces a form of identity disruption that re-entry to the workplace cannot automatically resolve. Service users in this study described absence in terms consistent with vocational identity collapse, uncertainty about professional competence, loss of role clarity and doubt about whether they belonged in working life at all. Therefore, RTW constitutes a process of identity transition rather than a simple logistical return. This is consistent with the role transition theory [43], where such transitions are seen as inherently uncertain, emotionally demanding and non-linear. This characterisation maps directly onto the fluctuating readiness, recurring self-doubt and setbacks participants described.

As one of the first studies to include EAs, our findings indicated that EAs act as a bridge between mental health services and employment and service user accounts highlight their significant role in facilitating RTW. It is possible that EAs provide an autonomy-supportive environment that addresses service users’ core needs for competence, relatedness and self-direction during the return process. This is consistent with the Self-Determination Theory (SDT) framework [44] and can further explain the motivational nature of personal drivers and integrated support that we observed. The findings also help reconcile the self-efficacy paradox- while quantitative research links high self-efficacy to successful RTW [11,17], in this study, participants described self-efficacy as dynamic rather than fixed, initially low (following absence) but gradually rebuilt through external support, positive feedback and small, manageable successes, each of which maps onto a core source of self-efficacy in Bandura and colleagues’ [45] framework: mastery experiences, verbal persuasion and improved physiological state respectively. This helps explain why self-efficacy appears both essential for RTW and simultaneously undermined by CMDs. Phased and graded returns operationalise mastery experiences (the “small successes” our participants valued); positive feedback from managers and EAs operationalizes verbal persuasion; and the affective nature of CMDs affects the physiological/affective source. In addition, RTW self-efficacy (RTW-SE) is an established, modifiable predictor [46] of return to work in employees with mental health problems. Critically, RTW-SE follows distinct trajectories over time. For example, Horn and colleagues [47] identified six RTW-SE trajectories, three of them increasing regardless of baseline (“class 1, low start, moderate increase; class 3, moderate start, small increase; class 5, moderate start, steep increase”); this resembles the rebuilding process our participants described. Because EA support, phased returns and managerial feedback each target a specific aspects of the self-efficacy framework as proposed by Bandura [45], our findings indicate that this framework could provide an actionable map for interventions. Together, these insights suggest that while the broad contours of CMD-related RTW are well established, the interplay between organisational structures, therapeutic and employment support and identity reconstruction is more complex than previously captured.

Taken together, these findings position RTW after CMDs as a multi-layered and emotionally complex transition that cannot be understood through organisational procedures alone. The interplay between individuals’ vulnerability, relational dynamics and structural conditions demonstrates that RTW is shaped as much by culture, communication and psychological safety as by formal policy. The study also highlights the importance of integrated support systems, showing that sustainable RTW depends on coordinated input across therapeutic, organisational and interpersonal domains. By bringing together HR, EA and service user perspectives, this research offers a more holistic account of RTW than is typically captured in single-stakeholder studies. This integrative understanding provides a foundation for identifying practical steps that organisations and services can take to support more sustainable and person-centred RTW processes.

### 4.1. Implications for Practice

The findings of this study highlight several practical implications for organisations, mental health services and policy makers seeking to improve RTW outcomes for individuals with CMDs. Participants consistently emphasised that rigid standardised procedures fail to accommodate the fluctuating nature of CMD recovery. Organisations should therefore move away from the one-size-fits-all approach and instead implement tailored RTW plans that adapt to individual needs, job demands and psychological readiness. This includes phased returns, temporary role modifications and flexible scheduling. Managerial behaviour emerged as one of the most influential determinants of RTW success. Many managers lacked confidence in discussing mental health, misapplied policies or avoided engagement altogether. Mandatory mental health literacy training, including communication skills, stigma reduction and practical guidance on adjustments, is essential. Organisations should also provide managers with structured tools like conversation guides and checklists to support consistent practice.

HR professionals in this study described being caught between policy constraints and employee needs. Their role should be reframed as facilitators of psychological safety and organisational learning, ensuring that policies are applied with empathy and contextual sensitivity. HR teams should also monitor patterns of managerial behaviour to identify training needs or organisational culture issues. Several practical strategies also emerge from the findings to help organisations foster and sustain the fragile self-efficacy following CMD-related absence. Phased and gradual returns are particularly valuable, enabling employees to experience small successes before re-engaging with the full demands of their role; these repeated opportunities for re-establishing competence are essential for rebuilding confidence incrementally. Graded RTW has been associated with significantly higher rates of sustained RTW (88% vs. 73%) and substantially fewer weeks of sick leave during follow-up (7.0 vs. 13.4 weeks) in employees with common mental disorders [48]. Relatedly, structured and regular feedback from line managers that emphasises progress and observable strengths, rather than deficits, can actively counteract the self-critical thinking that tends to dominate following prolonged absence [10]. Even though primary prevention of CMD is beyond this study’s scope, employers can still monitor psychosocial risk (e.g., unreasonable demands, poor role clarity) and adjust tasks or workload. Proactive measures include regular check-ins with returners, reasonable accommodations and promoting work–life balance. A supportive organisational culture (e.g., through employee assistance programmes or peer support groups) reinforces both well-being and retention. In addition, participants valued the combination of therapeutic input and employment support. Thus, timely, early referrals to EAs could potentially prevent long-term sickness absence and reduce the erosion of self-efficacy. A further recurring theme was the emotional impact of silence from employers during sickness absence. Organisations should establish clear communication protocols that maintain connection without exerting pressure, therefore helping employees feel valued rather than forgotten.

Lastly, employee–environment misalignment was a major barrier for RTW. Organisations should consider reviewing RTW policies to ensure they are easy to understand, flexible enough to accommodate individual differences and supported by training so managers can apply them confidently. Future longitudinal research tracking individuals over time to understand relapse risks, long-term job satisfaction, career progression and the durability of adjustments would be especially valuable to help us understand the impact of different interventions and processes on the above outcomes.

### 4.2. Strengths and Weaknesses

This study had several key strengths. Firstly, we were able to capture insights from three key stakeholder groups, service users, EAs, and HR professionals. The latter two groups have been particularly underrepresented in previous research; thus, this is one of the first studies which offers a convergence and divergence of barriers and facilitators from a multi-stakeholder perspective. Furthermore, we were able to recruit a diverse range of participants from different sectors and roles, thus enhancing the generalisability of our findings. In addition, we were able to reach data saturation, having interviewed beyond the typical recommendation of 12–15 participants for thematic analysis [49]. There are several limitations that need to be considered when interpreting the findings. All participants that were interviewed were UK-based; therefore, our findings may not reflect experiences in other cultural or organisational contexts. In addition, all participants were recruited within the wider London area, which may have affected the generalizability of our findings. Although, we did find convergence between the themes of this study and previous research, it is possible that geographic locations may have an impact on the participant experiences in relation to work. Therefore, further research encompassing wider geographic areas would be beneficial. Participants self-selected for this study; therefore, it is possible that individuals who volunteered may have had particularly strong experiences (either positive or negative) which could have influenced their participation. Lastly, interviewed participants were not connected in any way; triangulating data by recruiting triads of participants (an individual, their EAs and HR professional from the organisation) could potentially help us with capturing intricacies, nuances and subtle processes which affect the RTW journey.

## 5. Conclusions

This is one of the first studies which has investigated barriers and facilitators to RTW for individuals who have taken time off work due to CMD symptoms from multiple key stakeholders, namely individuals who have taken time off, employment advisors and HR professionals who support RTW journeys. Across these groups, several areas of strong convergence emerged, particularly around the centrality of managerial behaviour, psychological readiness and the need for flexible, person-centred support. However, notable divergences also surfaced: service users and EAs emphasised identity disruptions, emotional vulnerability and systemic pressures far more strongly than HR professionals, while HR accounts focused more on procedural readiness and policy applications. Therefore, this study demonstrates that returning to work after CMD-related absence is a complex, emotionally charged and relationally mediated process. HR professionals operated from within an institutional resource logic, offering procedural resources such as policies, phased return plans and formal review structures. Service users, however, most urgently required relational resources: acknowledgement, consistent contact, patience and empathic engagement. EAs occupied a distinctive bridging position, providing personalised guidance and confidence-building work that neither managerial nor HR frameworks typically offered. This tripartite dynamic of service user resource depletion, HR procedural provision and EA relational bridging represents a structural feature of CMD-related RTW that existing policy frameworks do not yet adequately account for. Ensuring that psychological, relational and identity dimensions of readiness carry institutional weight alongside procedural compliance is therefore as much a structural and cultural challenge as a training one. Successful RTW requires more than policy compliance, it depends on the alignment of organisational culture, managerial behaviour, psychological readiness and multi-layered support systems. By integrating HR, EAs, and service users’ perspectives, the study provides a holistic understanding of the structural and interpersonal dynamic that shapes RTW outcomes. The findings highlight the need for flexible, compassionate, and collaborative approaches that recognise the human dimensions of work, recovery and identity.

## Figures and Tables

**Table 1:** Summary of main themes and subthemes for barriers and facilitators of return to work following common mental disorder symptoms and sickness absence.

**Barriers**		
Level	**Theme** - Subtheme	Convergence/Divergence
Individual	**Identity disruption** - Reduced self-confidence and self-efficacy **Psychological Readiness** - Avoidance and fear	Service users and EAs emphasised confidence loss, identity disruption and threat anticipation. HR focused less on internal psychological processes and more on observable readiness.
Interpersonal	**Managerial Capability** - Limited mental health literacy - Lack of confidence (procedural and relational) **Managerial Disengagement** - Delayed or inconsistent communication **Psychological Safety** - Workplace stigma and social exclusion - Lack of empathy	All groups identified managers as central. HR framed gaps as training and policy issues, while service users described neglect or lack of empathy, and EAs highlighted limited mental health literacy. Service users foregrounded judgement and exclusion; EAs noted reduced rapport; HR acknowledged culture but less the lived impact.
Systemic	**Person–environment misalignment** - Organisational misalignment - Job misalignment **External environment misalignment** - Labour market constraints - Gaps in awareness and accessibility of support	All groups identified structural barriers. Service users and EAs additionally emphasised labour market pressures and access gaps, which were less prominent in HR accounts.
**Facilitators**		
Individual	**Personal drivers** - Reclaiming identity and purpose - Reframing setbacks	Service users and EAs highlighted motivation, identity and financial drivers. HR placed less emphasis on internal facilitators.
Interpersonal	**Managerial support** - Collaborative RTW planning - Awareness and empathy **Supportive relationships** - **Social inclusion**	Strong agreement on the importance of managerial support and communication. HR emphasised structured processes; service users and EAs stressed relational qualities (e.g., empathy, trust). Service users and EAs emphasised supportive relationships and normalisation. HR acknowledged relationships but less the role of informal and peer-based support
Systemic	**Tailored RTW support** - Phased and flexible RTW plans - Role clarity and alignment **Integrated support systems** - Access to support - Positive mental health attitude/resources	All groups agreed on the value of tailored RTW support. Service users and EAs further highlighted integrated support (e.g., EA/therapy), while HR focused on organisational implementation.

## Data Availability

The data presented in this study are available on request from the corresponding author.
